# Use of nested PCR for the detection of trichomonads in bronchoalveolar lavage fluid

**DOI:** 10.1186/s12879-019-4118-9

**Published:** 2019-06-10

**Authors:** Chao Lin, Furong Ying, Yanan Lai, Xiaolong Li, Xiangyang Xue, Tieli Zhou, Dongwei Hu

**Affiliations:** 1 The First Affiliated Hospital of Wenzhou Medical University, Wenzhou, 325000 Zhejiang Province China; 2 Wenzhou Medical University, Wenzhou, 325000 Zhejiang Province China

**Keywords:** Bronchoalveolar lavage fluid, Nested PCR, Trichomonad, Lung

## Abstract

**Background:**

The methods routinely used to detect trichomonads in the lungs are not sensitive enough, and an effective method is urgently needed.

**Method:**

Primers were first designed to match the conserved area of the 18S rRNA gene of trichomonads. Then, nested PCR was carried out to detect trichomonads in bronchoalveolar lavage fluid (BALF). Finally, all positive specimens were subjected to DNA sequencing and phylogenetic analysis.

**Results:**

Among 115 bronchoalveolar lavage fluid samples, ten samples tested positive in nested PCR (10/115), while no samples were positive in wet mount microscopy (0/115) (*P* < 0.01). Among the ten positive specimens, two were identified as *Tetratrichomonas* spp. and the other eight as *Trichomonas tenax* in phylogenetic analysis.

**Conclusions:**

Nested PCR is an effective way to detect trichomonads in bronchoalveolar lavage fluid.

## Background

Trichomonads are a type of parasitic flagellate protozoan of the genus that are found in the digestive and reproductive systems of man and animals. They frequently colonize the human lungs, but this condition is unfamiliar to most physicians [1, 2]. Several kinds of trichomonads can infect the lungs, such as *Trichomonas vaginalis, Trichomonas tenax, Pentatrichomonas hominis*, and *Tetratrichomonas* spp [3–6]. At present, microscopic detection is the most common approach for testing trichomonads in the clinic. However, numerous factors can make their recognition very difficult, if not impossible. [7, 8] First, immobility of trichomonads due to low temperature or for long periods in vitro can reduce the sensitivity. Second, many epithelial cells of lung alveoli move similarly to trichomonads, which could impede trichomonad detection [1, 9]. Third, trichomonads can develop into an amoeboid form, making them unrecognizable [10]. Therefore, there is an urgent need to establish a sensitive molecular method to diagnose pulmonary trichomoniasis. However, doing so is further complicated by the fact that numerous trichomonad species can infect human lungs. Existing gene based methods have been used to detect single types of trichomonads [4, 11–13]. However, not all species can be tested at a single time. To overcome this issue, all 18S rRNA gene sequences of different kinds of trichomonads were downloaded from NCBI, and then the most conserved area of this gene was identified through sequence alignment. Primers were then designed to cover this conserved region. To determine the prevalence of *Trichomonas* infection in the lungs, bronchoalveolar lavage fluids (BALF) from 115 cases were tested using nested PCR and microscopy. Furthermore, phylogenetic analysis was performed to determine which type of trichomonad is most likely to infect human lungs.

## Methods

### Bronchoalveolar lavage specimen collection

One hundred fifteen BALF samples were obtained from 113 patients, who visiting pulmonary specialists, at the First Affiliated Hospital of Wenzhou Medical University between 2017 and 2018 and were analyzed by investigators blinded to the clinical data. These BALF samples were obtained by fibre-optic bronchoscopy. When using bronchoscopy for alveolar lavage, oral contact is avoided as much as possible. Each patient was given 20 to 120 ml of normal saline irrigation based on the situation. Then, each sample was collected in 400 μl of sediment after centrifugation at 400 g for 5 min, after which the supernatant was discarded.

### Microscopy

Microscopy was utilized to detect trichomonads. A wet smear was created with 50 μl of sediment, followed by direct observation under a microscope. This direct microscopy was performed within two hours of BALF collection. Quality control of microscopy throughout the study was maintained by experienced microscopists who had more than 10 years’ work experience. All slides were rechecked within 15 min by experienced microscopists. Microscopy was performed at a magnification of 400×, and 20 fields were examined. The flagellated protozoa, which are as large as one to four white blood cells, were considered trichomonads. A negative diagnosis was made when no trichomonads were found.

### Genomic DNA extraction and polymerase chain reaction

Genomic DNA was extracted using a TIANamp Blood DNA Kit (Tiangen, China) from 200 μl of BALF sediment. The 18S rRNA gene was first amplified using the primers TRC1-F (5′-GGTAATTCCAGCTCTGCG-3′) and TRC1-R (5′-TGGTAAGTTTCCCCGTGT-3′). PCR was performed in a volume of 20.0 μl with 1.0 μl of DNA template, 0.3 μM of each primer, 2.0 μl of 10× PCR buffer, 2.0 units of DNA polymerase, and 0.2 mM dNTP mix. The reaction conditions consisted of initial denaturation at 98 °C for 2 min; 20 cycles of 98 °C for 10 s, 53 °C for 30 s and 68 °C for 30 s; and a final extension step for 5 min at 68 °C. Subsequently, the 18S rRNA gene was amplified by the primers TRC2-F (5′-GTTAAAACGCCCGTAGTC − 3′) and TRC2-R (5′-CCAGAGCCCAAGAACTAT-3′). PCR was performed in 20.0 μl with 0.4 μl of DNA template derived from the product amplified by TR1-F and TR1-R, 0.3 μM of each primer, 2.0 μl of 10× PCR buffer, 2.0 units of DNA polymerase, and 0.2 mM dNTP mix. The reaction conditions consisted of initial denaturation at 98 °C for 2 min; 35 cycles of 98 °C for 10 s, 54 °C for 30 s and 68 °C for 30 s; and a final extension step for 5 min at 68 °C.

### Sensitivity and specificity of the nested PCR assay

To compare the detection limit of the nested PCR assay with microscopy, serially diluted BALF containing *T. vaginalis* (0.1, 1, 10, 10^2^ trichomonads/μl) were used as the target, and all samples were analyzed in triplicate. The specificity of the nested PCR was assessed by testing the DNA samples from protozoa, including *T. vaginalis, T. tenax*, *T. hominis, Giardia intestinalis (G. lamblia)*, T*oxoplasma gondii and* human DNA*.*

### Molecular phylogenetic analysis

Through separation by agarose gel electrophoresis, a single amplicon of approximately 400 bp was obtained. After that, the amplicon was sequenced in both directions using the sequencing primers TRC2-F and TRC2-R. Finally, the sequences were used as queries for BLAST searches in the GenBank database (http://www.ncbi.nlm.nih.gov/Blast.cgi) to identify the types of trichomonads.

To further identify the species, the sequences were compared with 80 trichomonad 18S rRNA, which were acquired from the National Center for Biotechnology Information database. Multiple alignments were performed using the Clustal W program. The evolutionary history was inferred using the neighbor-joining method based on the Tamura-Nei model. [2, 14] The percentage of replicate trees in which the associated taxa clustered together was computed with a bootstrap test (1000 replicates) [15]. The tree was drawn to scale, with branch lengths in the same units as those of the evolutionary distances used to infer the phylogenetic tree. The evolutionary distances were computed using the maximum composite likelihood method [2] and are expressed in units of the number of base substitutions per site. The analysis involved 91 nucleotide sequences. The codon positions included were 1st + 2nd + 3rd + noncoding. All positions containing gaps and missing data were eliminated. There were a total of 314 positions in the final dataset. Evolutionary analyses were conducted in MEGA7 [16].

### Statistical analysis

One hundred fifteen samples were subjected to nested PCR and microscopic testing. The data were analyzed using Microsoft Excel and IBM SPSS 20.0. Fisher’s exact test was used to compare frequency data, and the Clopper-Pearson exact method based on the beta distribution was used to determine the 95% confidence intervals for proportions. Differences were considered statistically significant when *P* < 0.05.

## Result

### Detection results for different kinds of trichomonads

**Fig. 1:**
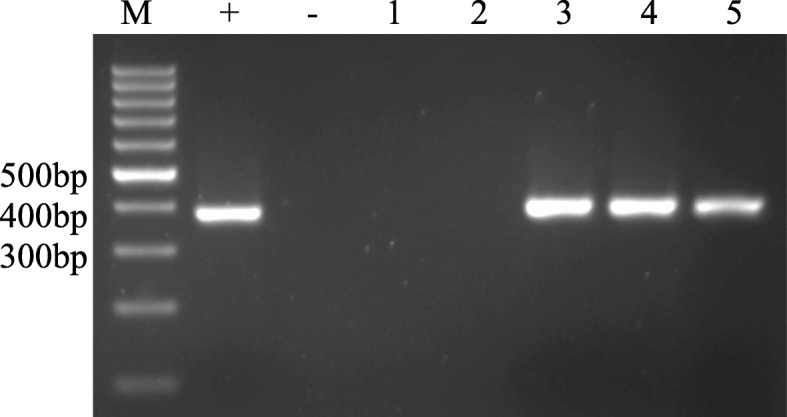
Detection of different trichomonads by nested PCR: GoldView-stained agarose gel showing the test results of trichomonad protozoa and other parasites. M, 100-bp DNA molecular size markers; +, positive control (*Trichomonas vaginalis*); −, negative control (human DNA); 1, *Toxoplasma gondii*; 2, *Giardia intestinalis*; 3, *Pentatrichomonas hominis*; 4, *Trichomonas vaginalis*; 5, *Trichomonas tenax*

To test the capabilities of the methods to detect trichomonads, the three most common trichomonads and two other common parasites in humans were tested. In gel electrophoresis after nested PCR, *T. vaginalis, T. tenax* and *T. hominis* displayed distinctive bands, while *G. lamblia,T. gondii* and the negative control, human DNA, displayed no band, and the positive control, a sample of *T. vaginalis*, displayed a distinctive band. (Fig. 1).

### Sensitivity of nested PCR

**Fig. 2:**
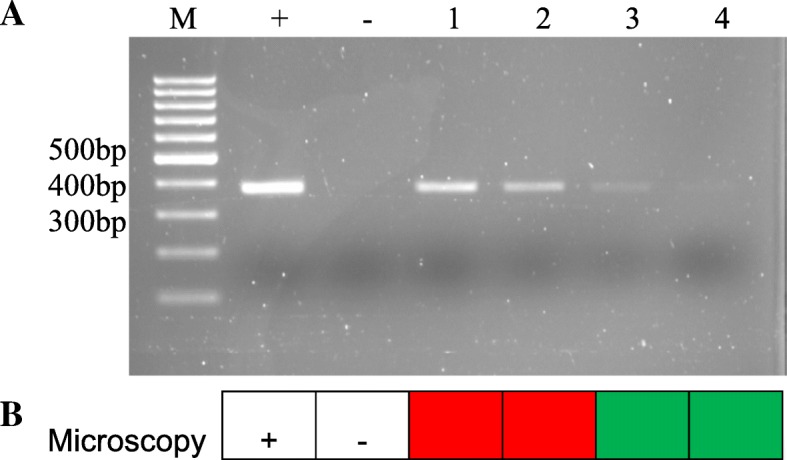
Assessment of the sensitivity of nested PCR for *T. vaginalis* in comparison with the microscopy test. **a** Sensitivity analysis of PCR assay on agarose gel. **b** Sensitivity analysis of microscopy. M, 100-bp DNA molecular size markers; +, positive control (*T. vaginalis*); −, negative control (human DNA). 1–4, Serial diluted BALF containing *T. vaginalis* (0.1, 1, 10, 10^2^ trichomonads/μl); the red square indicates positive results for microscopy; the green square indicates negative results for microscopy

The sensitivity analysis showed that the detection limit of the nested PCR assay was 100 trichomonads/mL of BALF (Fig. 2a). For microscopy, the detection limit was 10,000 trichomonads/mL of BALF (Fig. 2b).

### Test results for bronchoalveolar lavage fluid

**Fig. 3:**
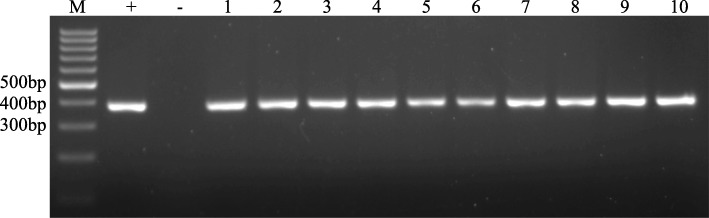
Gel image of positive samples: GoldView-stained agarose gel showing the PCR amplicons of the 18S RNA gene of *trichomonad species*; M, 100-bp DNA molecular size markers; +, positive control (*T. vaginalis*); −, negative control (human DNA); 1–10, positive samples

One hundred fifteen BALF samples were obtained from 113 consecutive patients who visited pulmonary specialists where investigators were blinded to the clinical data. The majority of patients were men (60%). The median age was 56 years (interquartile range, 47 to 66). Upon nested PCR analysis, 10 of the 115 specimens gave positive results. In addition, the negative control, human DNA, gave no band, while the positive control, *T. vaginalis*, had a distinctive band. (Fig. 3). None of the patients who tested positive were found to have a history of HIV infection or radiation treatment by retrospective analysis.

### Sequencing results

All PCR products of the ten positive samples were sequenced by a commercial organization using the TCR2-F and TCR2-R primers. The sequences were then used as queries for BLAST searches in the GenBank database (http://www.ncbi.nlm.nih.gov/Blast.cgi), and all sequences had no less than 99% homology (~ 380 bp) with *Trichomonas* sequences.

### Difference in positivity rates between the two methods

One hundred fifteen consecutive BALF samples obtained from 113 patients were tested by nested PCR and microscopy. Ten samples were positive in nested PCR, while none of the samples were positive in the microscopic test. (*P* < 0.01) (Table 1).

**Table 1:** Differences in positive rates between nested PCR and microscopy

	Positive (n)	Negative (n)	*P*
Nested PCR	10	105	
Microscopy	0	115	< 0.01

### Molecular phylogenetic analysis

**Fig. 4:**
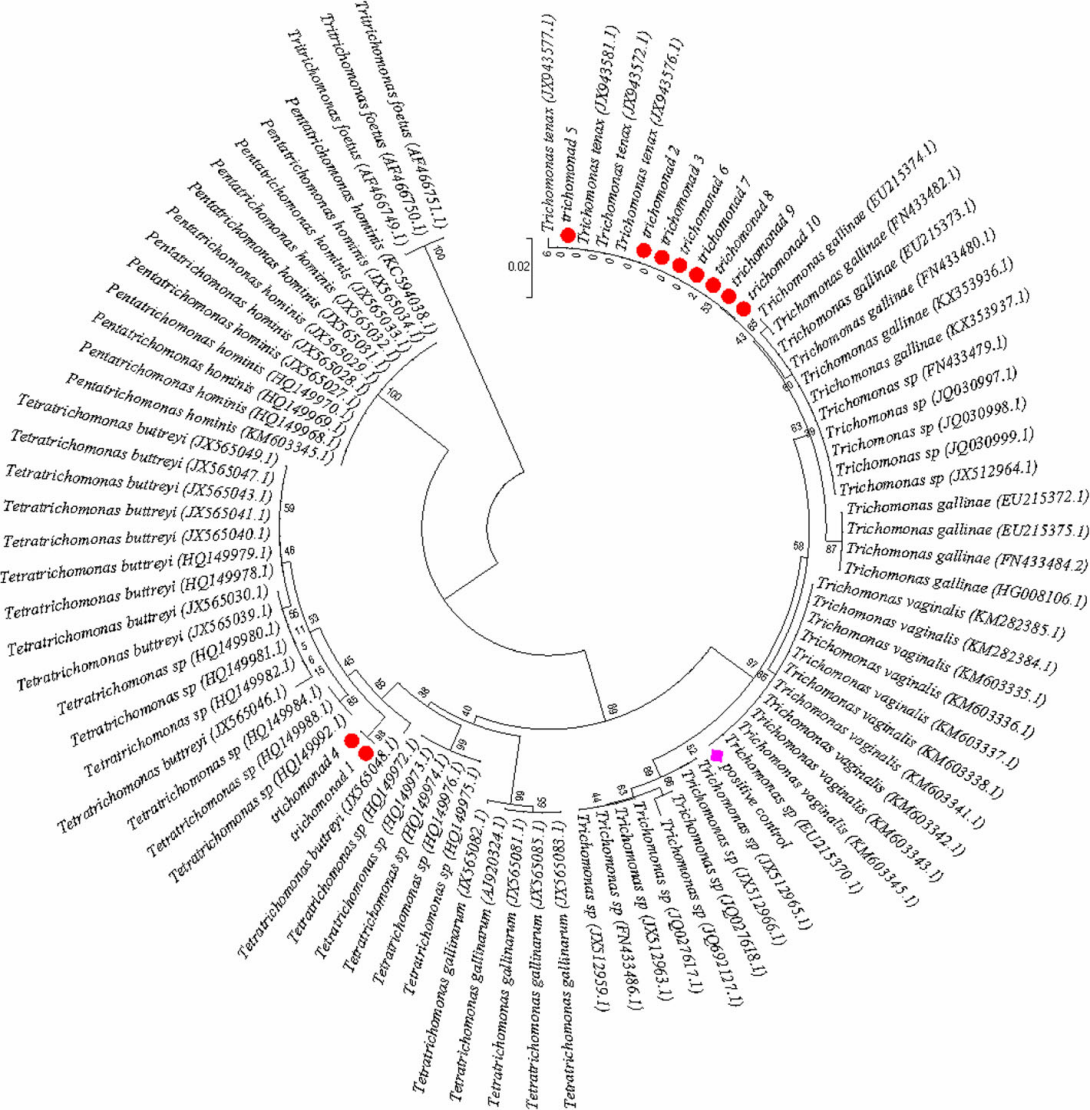
Evolutionary relationships of the trichomonads. Phylogenetic tree of several members of the family *Trichomonadidae* based on 18S RNA sequences. The percentages of replicate trees in which the associated taxa clustered together in the bootstrap test (1000 replicates) are shown next to the branches. The sequences of the ten positive specimens, shown by the red dots in the figure, were compared with 80 trichomonad sequences available in the National Center for Biotechnology Information database. The GenBank accession numbers are shown in parentheses. *T. vaginalis,* shown by a pink rhomboid in the figure, was used as a positive control and was grouped in the appropriate species clades

The sequences of the ten positive samples were compared with 80 trichomonad 18S RNA sequences acquired from the NCBI and a positive control. The analysis involved 91 nucleotide sequences. The codon positions included were 1st + 2nd + 3rd + noncoding. All positions containing gaps and missing data were eliminated. There were a total of 314 positions in the final dataset. Evolutionary analyses were conducted in MEGA7 [16]. As shown in Fig. 4, two samples were classified as *Tetratrichomonas*, and the others were classified as *Trichomonas tenax.* (Fig. 4).

## Discussion

In most respiratory tract infections, the causative pathogen remains unknown, so there is an urgent need to improve detection methods. Trichomonads are a type of protozoan with several flagella, and they are microscopic in size. [17–19]. Thus, trichomonads cannot be directly observed by the naked eye. Microscopy is still routinely used to detect trichomonads. [19]. However, it is sometimes difficult to distinguish trichomonads for several reasons. First, some normal ciliated columnar epithelial cells can move like protozoa in wet films. Second, the BALF collected from patients infected by trichomonads always contains many inflammatory cells, which will interfere with the detection of trichomonads. Third, trichomonads may lose mobility if not detected in a timely manner. Therefore, it is sometimes difficult to accurately detect trichomonads with routine methods in the clinic.

Our studies have shown that the nested PCR that we designed can detect trichomonads in BALF specimens in a sensitive and specific manner. Among the 115 BALF samples analyzed, ten samples were positive in nested PCR (10/115), while none of the samples were positive in a wet microscopy test (0/115) (*P* < 0.01). (Table 1) The positivity rate of nested PCR was significantly higher than that of microscopy. Moreover, nested PCR was very specific. Upon BLAST searches in the GenBank database (http://www.ncbi.nlm.nih.gov/Blast.cgi), all of the sequences revealed no less than 99% homology (~ 380 bps) with *Trichomonas* sequences.

Moreover, our study suggests that an unidentified *Tetratrichomonas* sp. and *T. tenax* are very important species among the trichomonads that can infect humans. Similar to previous studies, we also showed that *Trichomonas tenax*, a common inhabitant of the human oral cavity, is the most common species to infect human lungs [3, 20]. *Tetratrichomonas* spp. are usually reported as pathogenic agents in animals rather than in humans [21, 22]. In our study, however, two of the ten positive samples were identified as *Tetratrichomonas* spp. Whether these *Tetratrichomonas* spp. can spread between humans and animals need further confirmation. Further studies should seek to elucidate the mechanisms of the transmission and pathogenesis of these *Tetratrichomonas* spp.

One limitation of our study is that we cannot be sure that this nested PCR method can detect trichomonads for which no 18S RNA sequences exist in GenBank.

## Conclusion

In conclusion, the results of our study demonstrate that nested PCR is an effective way to detect trichomonads in BALF. Moreover, our study suggests that *Tetratrichomonas as well as T. tenax* is a very important species among the trichomonads that can infect humans. Our findings support the application of nested PCR in the detection of trichomonas in the clinic. Further research in this area should seek to elucidate the mechanisms of the transmission and pathogenesis of *Tetratrichomonas* spp.

## Data Availability

The datasets used and/or analyzed during the current study are available from the corresponding author on reasonable request.

## Abbreviations

BALF: Bronchoalveolar lavage fluid
NCBI: National Center for Biotechnology Information database
PCR: polymerase chain reaction
